# Editorial: Over 60 years of neurochemistry, the heritage of Dr. Ricardo Tapia

**DOI:** 10.3389/fnmol.2024.1398127

**Published:** 2024-03-26

**Authors:** Josué Orlando Ramírez-Jarquín, Angeles C. Tecalco-Cruz, Violeta Gisselle Lopez-Huerta, Uri Nimrod Ramírez-Jarquín

**Affiliations:** ^1^Departamento de Neuropatología Molecular, Instituto de Fisiología Celular, Universidad Nacional Autónoma de México (UNAM), Mexico City, Mexico; ^2^Posgrado en Ciencias Genómicas, Universidad Autónoma de la Ciudad México, UACM, Mexico City, Mexico; ^3^Departamento de Neurodesarrollo y Fisiología. Instituto de Fisiología Celular, Universidad Nacional Autónoma de México (UNAM), Mexico City, Mexico; ^4^Departamento de Farmacología, Instituto Nacional de Cardiología Ignacio Chávez, Mexico City, Mexico

**Keywords:** Ricardo Tapia, neurodegenerative diseases, spinal cord damage, ischemia, amyotrophic lateral sclerosis, brain injury, reactive oxygen species, mitochondrial death

Professor Ricardo Tapia was one of the most notable Mexican scientists (Figure 1), who began his scientific career around 1960 by working with his mentor, Dr. Guillermo Massieu. Later Dr. Tapia continued his prominent career by joining for postdoctoral instruction to Dr. Jorge Awapara's laboratory in Houston, Texas, and later to the group of Dr. Robert Balazs in London. Along this journey, he acquired significant knowledge and expertise in the GABAergic system of the CNS, emerging as a pioneer in his field.

**Figure 1:**
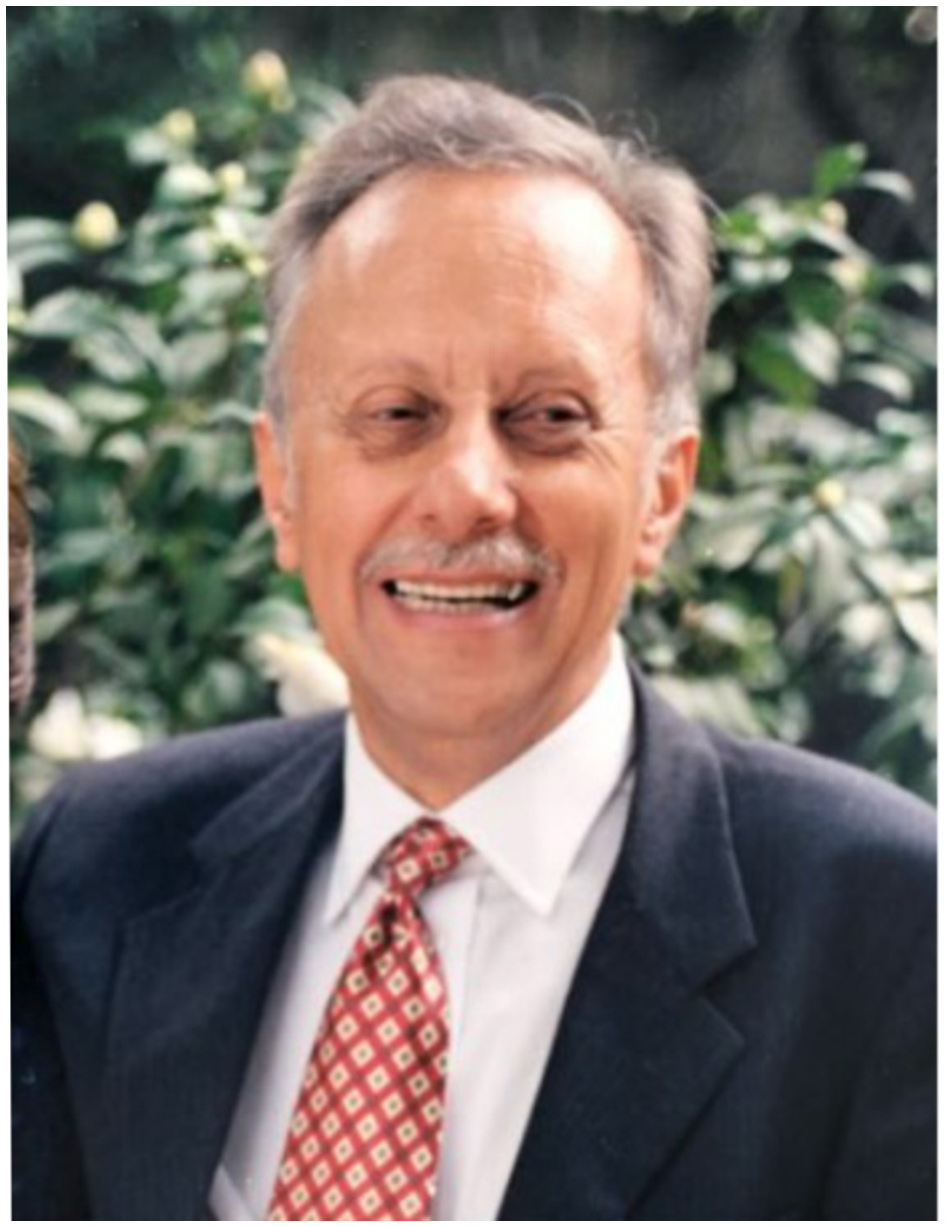
Dr. Ricardo Tapia. Image source: Maru Perez. Reproduced with permission.

Upon his return to Mexico in the late 1970's, he was incorporated into the National Autonomous University of Mexico (UNAM) and established his own research group. Since then, Professor Tapia was tremendously active in science as well as in several academic activities. He was the founder of centers and institutes for research, as well as bachelor's, Master's and Doctorate programs focused on science, and bioethics. Over more than 50 years as a leader in neurochemistry of the CNS, Professor Tapia published 158 scientific articles, got more than 6,000 citations, and more than 60 undergraduate, master's, and doctoral students in his lab. Notably, a significant portion of his students are still engaged in scientific research, contributing to knowledge in diverse areas (one of the most important aspirations for him). Some of the research areas explored by Professor Tapia's alumni are included in this special topic which contain six manuscripts (four original papers and two reviews), covering themes, such as, neuronal death, ischemia, neurodegenerative diseases, mitochondrial death, spinal cord damage, and amyotrophic lateral sclerosis.

The review by García-Velázquez and Massieu covers molecular mechanisms involved in neurodegenerative diseases, emphasizing the importance of ketone bodies (KBs), particularly β-hydroxybutyrate, in physiological conditions and as a possible therapy for the treatment of some neurodegenerative diseases. The authors discuss the importance of KBs, in metabolism for energy, as well as their role in cellular mechanisms such as inflammation, ROS production and mitochondrial protection, and atherosclerosis. They also include the epigenetics mechanisms of β-hydroxybutyrate for post-translational modifications (β-hydroxybutyrylation of histones, specifically in lysine residues) and as an inhibitor of HDAC. The control mechanism by which KBs regulate cellular proteostasis, by controlling the Unfolded Protein Response (in a context-dependent manner), and promoting autophagy through AMP-activated protein kinase activation, can be associated with the application of KBs, ameliorating the alterations observed in Alzheimer's Disease (AD) and Parkinson's disease (PD).

In their review, Acosta-Galeana et al. compared the mechanisms between multiple sclerosis (MS) and amyotrophic lateral sclerosis (ALS) in different experimental models. This work addressed the relevance of energy balance and cellular transport in the regulation of processes such as RNA binding proteins mislocalization, misfolding proteins and aggregation, mitochondrial dysfunction, proteostasis, granule stress, hyperexcitability, and axonal genesis alterations. The authors also highlighted the impairment of TDP-43 (Transactive response DNA-binding Protein 43) and hnRNP A1 (heterogenous Ribonucleoprotein A1) in both diseases. Some other mechanisms of interest in this work were inflammation in ALS, immune response in MS, neurodegeneration, and peripheral disturbances.

At the cellular level, the original paper by García-Hernández and Morán explored the effect of ROS induced by staurosporine or low potassium conditions on the survival of cerebellar granule neurons. They found that these conditions increased the expression of TXNIP (Thioredoxin Interacting Protein), which is required for cell death processes. Through ChIP experiments, they demonstrated that TXNIP overexpression is promoted by FOXO 3 activation, through Akt inactivation. These data denote the fundamental role of ROS, a common feature of different neurodegenerative conditions.

Neurodegenerative disorders research was also part of this topic. AD mechanisms and treatment were the theme of the work for Robles-Gómez et al.. Using a transgenic mouse (sTg4510, which mimics tauopathies) with or without intracerebroventricular administration of β-amyloid, they found that epothilone-D (a microtubules stabilizing agent) in an AD context prevents memory impairment in this model, even in presence of β-amyloid. Neuronal disorders such as the attention deficit/hyperactivity disorder were included too, Vázquez-González and Corona evaluated the effect of pioglitazone, an agonist of the nuclear receptor PPARγ (peroxisome proliferator-activated receptor), in an experimental model of this disorder, induced by 6OHDA administration, and characterized by hyperactivity after 6OHDA treatment. The authors demonstrated that motor activity was not affected by pioglitazone, but they observed an increased expression of genes involved in mitochondrial biogenesis, such as SDHA, COX-I, PGC1α, and the transcription factor Nrf2 in different brain structures.

Novelty and innovation of procedures were covered by the work of Morales-Villagrán et al., who designed a novel mode for brain trauma injury. Current protocols for brain injury use fluid percussion, which requires a craniotomy to apply a pulse of fluid on the dura to generate a distortion of brain tissue and cause neurological damage. One important consideration in this procedure is the pressure variation that happens during the fluid percussion. In their study, Morales-Villagrán et al., used a solenoid to control and keep a constant pressure of the wave over the dura of adult rats, to validate the relevance of their protocol, the authors performed sensorial (general balance, landing test, tail raise test, drag test, righting reflex), motor (ear, eye, sound, tail, and paw flexion reflexes), and cognitive tests (Morris Water Maze), as well as histology for tissue biomarkers of damage. Summarizing their evidence, the authors conclude that constant pressure controlled by solenoids are effective to induce brain injury.

Professor Tapia's academic legacy extends across several research areas, some of them included in the present topic, honoring his tireless dedication and love for science, now carried on by his alumni.

## Author contributions

JOR-J: Writing—original draft, Writing—review & editing. ACT-C: Writing—review & editing. VGL-H: Writing—review & editing. UNR-J: Conceptualization, Project administration, Writing—original draft, Writing—review & editing.

